# Effect of Laser Speed and Hatch Spacing on the Corrosion Behavior of 316L Stainless Steel Produced by Selective Laser Melting

**DOI:** 10.3390/ma15041353

**Published:** 2022-02-12

**Authors:** Antonio Collazo, Raúl Figueroa, Carmen Pérez, Xosé Ramón Nóvoa

**Affiliations:** CINTECX, ENCOMAT Group, Campus Universitario As Lagoas, Universidade de Vigo, Marcosende, 36310 Vigo, Spain; acollazo@uvigo.es (A.C.); cperez@uvigo.es (C.P.); rnovoa@uvigo.es (X.R.N.)

**Keywords:** SLM, 316L, corrosion, impedance

## Abstract

In this work, the corrosion properties of 316L stainless steel (SS) obtained by selective laser melting (SLM) are analyzed. The electrochemical results of samples manufactured with an energy density between 40 and 140 J/mm^3^ are compared using different hatch distances and laser speeds. The analysis correlates the impact of the microstructure and processing defects of SLM 316L stainless steel on its behavior against corrosion. The optimal manufacturing conditions were selected considering the electrochemical results. Although the samples obtained with an energy density close to 90 J/mm^3^ show a high resistance to corrosion, their performance depends on the combination of selected parameters, obtaining the best results for an intermediate laser speed and a low hatch distance. These manufacturing conditions produce a higher breakdown potential, a faster repassivation of the steel and reduce the current density on electrochemical test.

## 1. Introduction

Additive manufacturing (AM) is an attractive technique that makes it possible to create complex free-form objects with a 3D model by using additive manufacturing technology. Traditionally, the AM process was used to create visualization model products, but this technology developed over time, improving the properties, accuracy, and overall quality of materials, allowing suitable outputs for end use [1].

AM has a number of advantages, such as the possibility of manufacturing complex parts, efficient use of materials without subsequent machining, suitability for low production volumes, manufacturing with a wide variety of metal alloys, and the search for new alloys. There are different AM techniques that can be included in the following methods: fused deposition modeling (the most common used for polymer filaments), powder bed fusion, inkjet binding and contour crafting, stereolithography, direct energy deposition and laminated object manufacturing [2,3]. The advantages, disadvantages, fields of application and materials used in each method can be found in the review of Ngo, T.D et al. [2] 

The SLM technique used in this study belongs to the powder bed fusion method. In SLM, specimens are manufactured using laser scanning, which selectively melts and joins successive layers of powder [4,5]. The build parameters employed (laser power, scanning speed, layer thickness, laser scanning path, hatch space, powder size, etc.) produce substantial changes in sample properties and their optimization is essential for obtaining samples with adequate properties. The SLM process involves a rapid heating and fast cooling rate [6], which result in unique microstructures with refined grain structures [7]. The process also generates metallurgical defects such as a lack of fusion, porosity by entrapped gas, dislocation networks, residual stress, solute segregation, surface roughness, among others, which can result in worse properties than traditional manufacturing methods [7,8,9].

In the recent years, the AM of AISI 316L has been extensively studied. Although it is a steel for applications where a high resistance to corrosion is necessary, most of the studies are based on the assessment of the microstructural and mechanical properties [10,11,12,13,14,15,16,17]. The microstructure of AM 316L SS consists of very fine interconnected cellular or columnar sub-grains inside each individual large grain of austenite (δ-ferrite may be present in intercellular regions) [18]. The optimal AM conditions can produce near-full density samples with a higher tensile strength and ductility than those produced via conventional manufacturing processes [13,19]. 

With regard to corrosion resistance, there is also a wide variety of studies on AM 316L, although the results are not conclusive. As occurs in the mechanical performance, the corrosion resistance behavior is highly conditioned by the manufacturing conditions. Table 1 summarizes some of the most representative corrosion studies of SLM 316L SS and the analyzed manufacturing parameters. Some of these studies indicate that AM improves the corrosion resistance of SS compared with wrought samples [20,21,22,23,24,25,26,27,28,29], although similar behavior has also been reported [30,31], with even worse performances for SLM 316L SS [32,33,34,35]. The higher corrosion resistance is associated with the rapid cooling of AM, which reduces nucleation and crystal growth. This produces a homogeneous distribution of alloying elements, avoiding the formation of chromium-depleted regions and reducing inclusions such as MnS, minimizing the nucleation of pits [12,20,21,32]. The worst corrosion resistance has been associated with the presence of small amounts of δ-ferrite, microsegregation and a high level of porosity [22].

Despite all of these studies, it is not clear which are the best manufacturing conditions to produce the optimal corrosion resistance properties, since the works with the best results only partially indicate the manufacturing conditions or uniquely show the results for a fixed condition. For this reason, more studies are necessary to deepen the knowledge of AM and to improve the corrosion resistance properties of specimens. The present study aims to evaluate the corrosion behavior of SLM 316L SS building with different input energy density, hatch spacing and traverse speed. Several conditions were compared to select the optimal manufacturing parameters.

## 2. Materials and Methods

**Figure 1:**
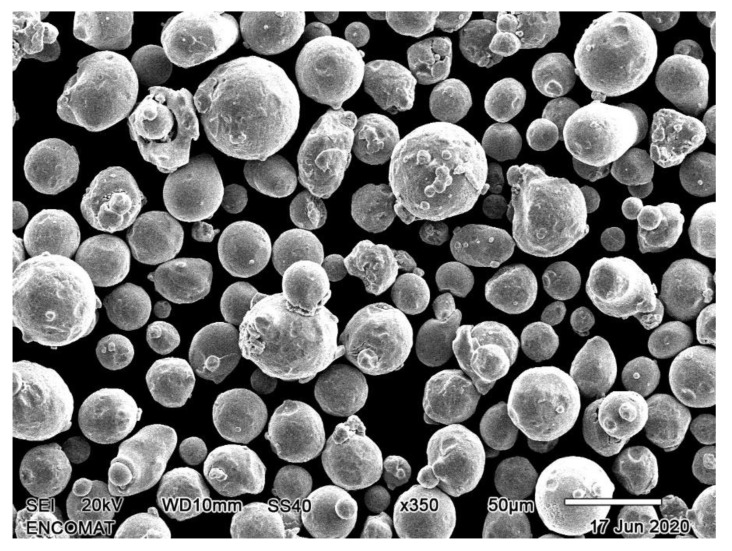
SEM micrograph of 316L stainless steel powder provided by the EOS GmbH (Germany).

The powder used for the SLM builds was EOS stainless steel 316L. The chemical composition corresponds to ASTM F138 material standard (UNS S31673). The particle size is in the range between 5 and 40 μm, and its chemical composition is shown in Table 2 as indicated in the material data sheet provided by the EOS GmbH (Germany). A micrograph of the powder is depicted in Figure 1, where the spherical morphology and size distribution of the powder particles can be observed. Additionally, for a comparative purpose a commercial wrought 316L SS with a similar composition was used. 

SLM 316L SS were manufactured by 3D printer EOS M290 equipped with a 400 W fiber laser. Cubic samples with 10 mm × 10 mm × 10 mm dimensions were produced, keeping the laser power (370 W) and the layer thickness (0.04 mm) constant. The scanning strategy followed a zigzag pattern with 67° laser beam rotation between each of the layers. The hatch distance and the laser speed were varied in order to change the processing conditions. The processing parameters are listed in Table 3. The samples are labeled in alphabetical order with a letter and the energy density used in their manufacture (rounded value).

The energy density *E (*$\frac{J}{mm^{3}})$ shown in Table 3 was calculated by taking into account Equation (1), where *P* is the laser power (W), *v* is the scanning speed (mm/s), *h* is the hatch distance (mm) and *t* is the layer thickness (mm) [36]:

(1) $$E=\frac{P\;}{v\cdot h\cdot \;t}\;$$

Cubic samples were cut to analyze the cross section parallel to the build direction (i.e., XZ-plane). Before microstructure examinations and the hardness and corrosion tests, all samples were subjected to the metallographic preparation: grinded with successive grades of SiC papers up to 1200 and polished with diamond suspensions (6 µm, 3 µm and 0.04 μm (colloidal silica)). Afterwards, the samples were degreased ultrasonically in acetone for 5 min, rinsed in water and dried.

The porosity of specimens was analyzed from several optical images of polishing surface at different magnifications using the ImageJ software 1.53k version (MD, USA) [37]. To reveal the microstructure, the specimens were etched in 5 mL HCl, 1 g picric acid, and 100 mL 95% ethanol (Vilella’s reagent). The microstructure was characterized by optical microscopy (Olympus GX51) equipped with an Image Analysis software from Olympus® (Olympus Corporation, Tokyo, Japan) and a scanning electron microscopy (SEM) model JEOL 5410 equipped with an energy dispersive spectroscopy probe (EDS) from OXFORD® instrument (Abingdon, England). 

Electrochemical experiments were performed at room temperature in 3.5 wt% NaCl solution, using a conventional three-electrode cell, where the working electrode was 316L stainless steel (0.5 cm^2^ of exposed area). A graphite sheet was used as counter electrode and a saturated calomel electrode (SCE) as reference electrode. An AUTOLAB 30 Potentiostat with FRA module (from EcoChemie^®^, Metrohm AG, Herisau, Switzerland) was used for potentiodynamic and electrochemical impedance measurements. After 3600 s stabilization period, the electrochemical impedance spectra were recorded at the open circuit potential (OCP) from 100 kHz down to 10 mHz, at 10 mV rms signal amplitude. Then, the cyclic potentiodynamic polarization was started at a cathodic potential at −0.05 V vs. OCP, scanning up to 1.2 V vs. SCE (with a scan rate of 1 mV/min) and with a limiting current density of 1 mA/cm^2^ to avoid overgrowth of pits. Subsequently, the scan was reversed in the anodic direction down to −0.5 V vs. SCE and continued in a forward direction up to the initial potential. To verify the reproducibility of the obtained results, at least three replications were made for each specimen.

Vickers micro-hardness measurements (HV0.2) were performed on the cross section of the samples with an ENCOTEST DuraScan microhardness tester (Emco-test Prüfmaschinen GMBH, Kuchl, Austria) according to UNE-EN-ISO 6507-1:2006. The hardness value for each material was the average value of 30 indentations in parallel directions to the building direction. 

## 3. Results and Discussion

### 3.1. Microstructural Characterization 

**Figure 2:**
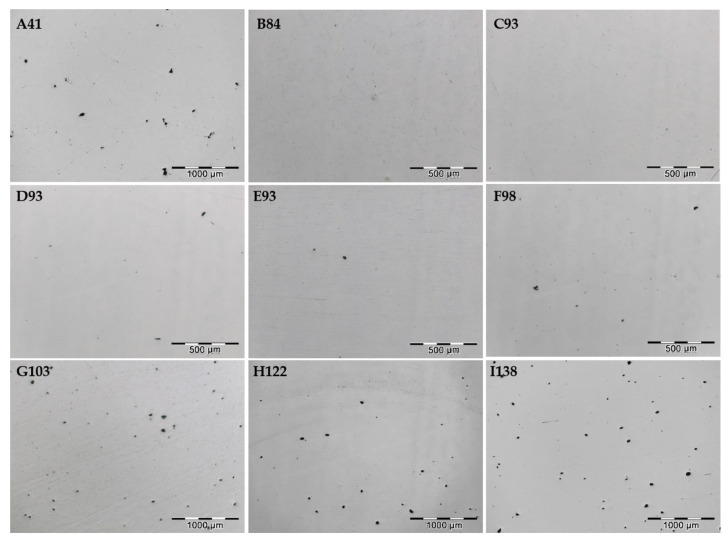
Optical micrographs of the polished surface of SLM samples showing the porosity.

Figure 2 shows the optical micrographs of the polished XZ plane of different SLM 316L SS samples. The presence of porosity can be clearly seen in most samples. However, it is worth highlighting the low porosity of samples B, C, and D obtained with an intermediate energy density, close to 90 J/mm^3^. The porosity of the samples was estimated based on several optical micrographs from each sample by ImageJ software. This analysis was not performed on a micrometer scale, since the impact of submicron pores in determining porosity is numerically negligible [23]. 

Table 4 displays the porosity values, which are less than 0.7% for all samples, and even below 0.2% for samples C93 and D93. Two types of pores can be identified in the micrographs: irregular and spherical. Irregularly shaped pores appear in SLM at the melt pool boundaries due to lack of fusion. The gas between powder particles is not entirely released. This porosity is produced when the low energy density or insufficient overlap between the scan tracks are used [6,24], and can be easily identified in the sample A41, which shows large pore sizes of up to 150 µm and are irregular in shape. Spherical pores appear inside the melt pools and are generated due to the entrapment of gases during the solidification due to the high energy density [6,16,24]. Spherical porosity is present in all SLM samples, although it is especially important in the samples H122 and I138, which were obtained with a higher energy density, and where a high amount of pores with diameters up to 50 µm can be appreciated.

**Figure 3:**
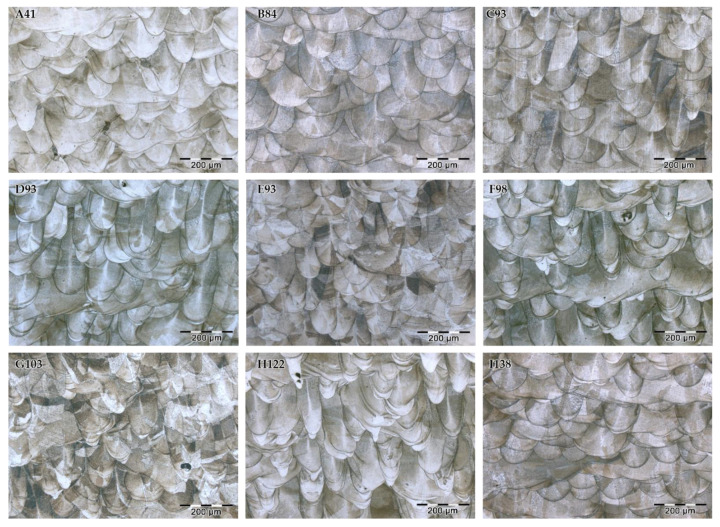
Optical micrographs of the cross section (XZ plane) of SLM samples after etching.

**Figure 4:**
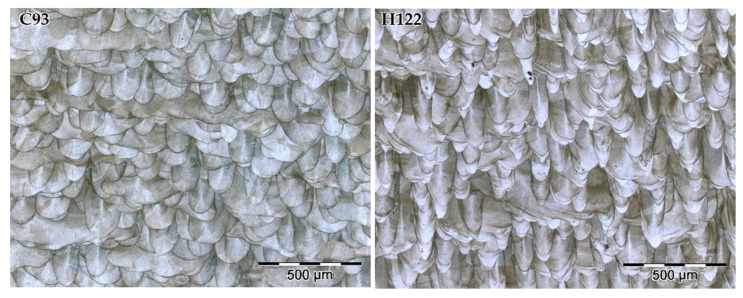
Optical micrographs of the cross section (XZ plane) of C93 and H122 SLM samples after etching at ×100 magnification.

Figure 3 shows the optical micrographs of the XZ cross section of SLM 316L SS specimens after metallographic etching to reveal their microstructure. A complex network of overlapped melt pools is clearly identified in all samples. Sample A41 exhibits the more irregular melt pools and large defects due to the lack of fusion. The other SLM samples present a similar microstructure with well-defined melt pools and without qualitative differences. The cut of the cubic specimens produces large differences in the size of the melt pool cross section because of the laser-scanning pattern used, with angle variations in each layer. Taking this into account, it can be noted that the samples with more homogeneous microstructures are C93 and D93. The sample H122 shows the narrowest and deepest melt pools. This pattern can be seen more clearly by comparing the micrographs at a lower magnification, as shown in Figure 4. The morphology of the melt pools for sample H122 is produced by the “keyhole-mode” melting mechanism [38]. Keyhole-mode laser melting is more likely at high energy density and low speeds and results in a trail of voids in the wake of the laser beam [39]. Therefore, it is expected that sample H122 shows low corrosion resistance.

**Figure 5:**
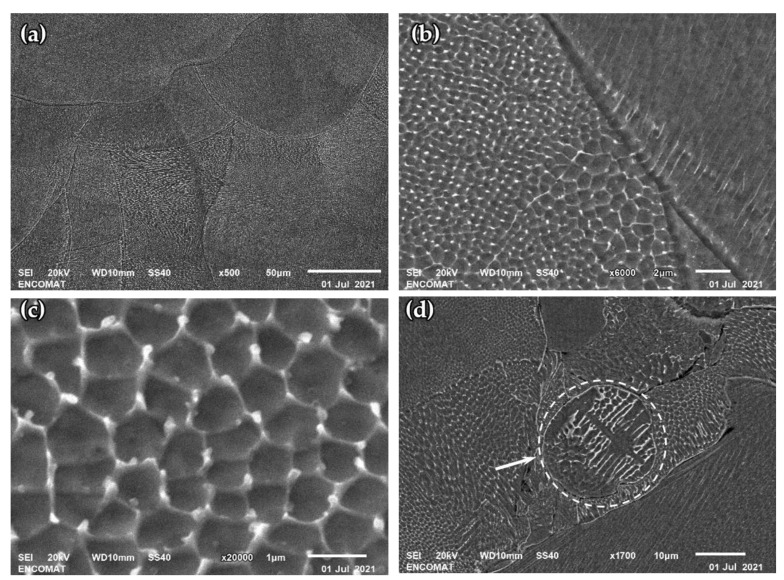
(**a**–**c**) Representative SEM images of the etched SLM 316L SS samples show the cellular and columnar structures at different magnifications. (**d**) SEM micrograph of A41 sample shows unmelted particle with dendritic microstructure.

The microstructure characterization of SLM specimens by SEM clearly reveals the melt pools with cellular and columnar substructures, as can be seem in Figure 5a. This morphology has been reported previously by several authors [14,25,26,35]. Additionally, in Figure 5d, which corresponds to sample A41, the presence of non-melted powder particles can be observed. The unmelted particles exhibit a dendritic microstructure, which is completely removed after laser fusion. In the specimens manufactured with a higher energy density, no unmelted particles were found. At higher magnifications, the cellular and columnar morphology present in the interior of the melt pools can be seen more clearly (Figure 5b,c). The size of cellular structure is highly variable, from 0.5 up to 2 µm [25,35]. This variability is related to the high temperature gradients and different solidification rates produced by the rapid movement of the laser beam [14]. Due to this variability, no significant differences were found between microstructures of SLM specimens.

The contrast between the cell boundary and its interior indicates a higher resistance of the boundary to the etching solution, suggesting a different composition [40]. The difference is attributed to the microsegregation of Mo and Cr [14,35], although several works have not found composition differences between both zones [9]. Considering the work of Rännar et al. [40], where a thickening of the cell boundary is observed for a lower cooling rate, the difficulty in identifying microsegregation is most likely due to the different manufacturing conditions. Rapid cooling produces a slight microsegregation and makes its analysis difficult. In our case, we were not able to identify differences in the composition of both zones by EDS.

### 3.2. Hardness Measurements

Although the mechanical characterization of the samples is not the objective of the present work, microhardness measurements were carried out to check whether the selection of the parameters that give rise to a better corrosion resistance also produces a higher hardness of the samples. In addition, hardness measurements can also determine the mechanical properties heterogeneity of the samples.

**Figure 6:**
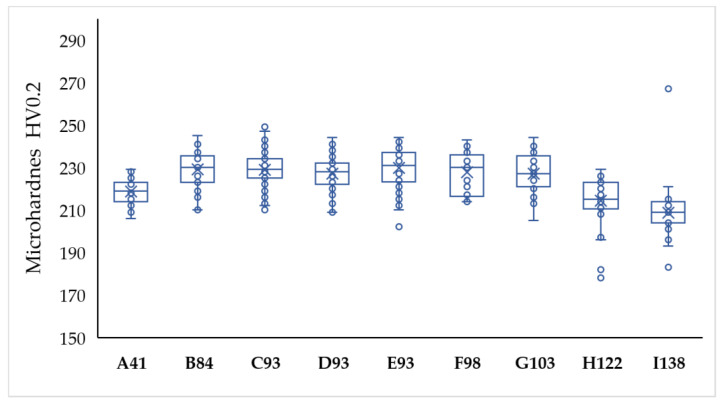
Whisker plot of microhardness for the SLM 316L SS.

Figure 6 depicts a whisker plot of the microhardness values measured parallel to the building direction. Hardness was also measured perpendicular to the manufacturing direction, but no differences were found [10].

All SLM samples, with the exception of A41 H122 and I138 samples, show hardness values close to 230 HV, and the statistical analysis of the results indicates that there are no differences between them. These results are similar to those of other AM of 316L studies with hardness values between 210–240 HV [10,41,42,43]. The lower hardness values of samples manufactured with low and high energy densities (A41 H122 and I138) is due to their greater porosity, which yields the collapse of the material under load, also producing a greater deviation in the measurements [42].

### 3.3. Electrochemical Analysis 

**Figure 7:**
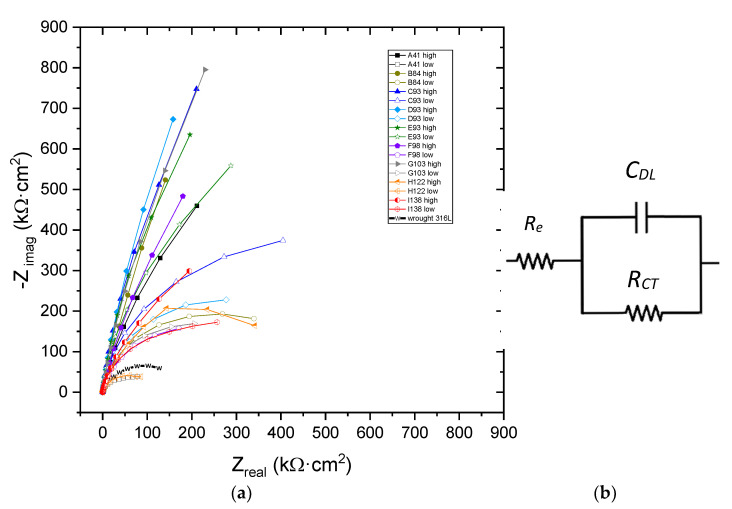
(**a**) Experimental Nyquist plots obtained for SLM 316 SS specimens after one hour of immersion in 3.5% NaCl solution (maximum and minimum values are displayed for each sample). (**b**) The EEC model used to simulate the impedance spectra.

The impedance measurements were carried out after 1 h of immersion in the 3.5%wt. NaCl solution to stabilize the OCP and reach equilibrium. Despite the stabilization period, it was not possible to obtain a good repeatability of the impedance measurements to adequately analyze the results. A minimum of five impedance measurements were performed for each sample. Figure 7a depicts the Nyquist plots obtained for SLM 316L SS samples and wrought 316L SS. Two curves are included for each SLM sample corresponding to the maximum and minimum impedance values measured.

As can be seen, all Nyquist plots exhibit one capacitive loop. As expected, the samples that present a higher impedance correspond to those manufactured with intermediate energy density. Lower impedance values were obtained for A41, H122 and I138 samples. Therefore, the high variability of the impedance values is related to the porosity present in the AM samples [30]. As indicated before, all samples present porosity, and so the high variability is consistent with the impedance results, since EIS is very sensitive to small defects present on the surface or pits that can be activated during the measurement. The pores can be very close to the surface so that the ingress of electrolyte during the stabilization period can significantly reduce the measured impedance.

The impedance results were fitted using an equivalent electrical circuit (EEC) depicted in Figure 7b. This EEC has already been used by other authors for 316L SS [26,44]. In this model, *R_e_* is the solution resistance, *C_DL_* the double layer capacitance, and *R_CT_* the charge transfer resistance. The impedance (*Z*) of the proposed EEC is given by Equation (2), where ω = 2πf, *j* = −1^1/2^ and α is a Cole–Cole parameter, which accounts for the dispersion of the time constant:

(2) $$Z=R_{e}+\frac{R_{CT}}{1+{\left(jwR_{CT}C_{DL}\right)}^{\alpha }}$$

**Figure 8:**
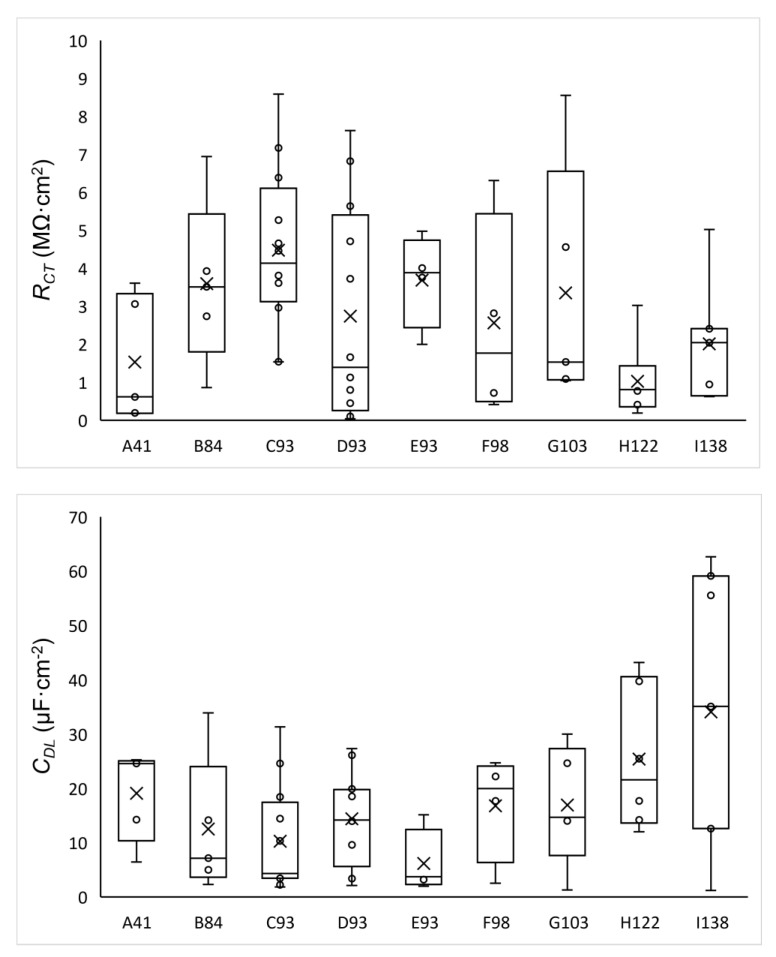
Whisker plots of *C_DL_* and *R_CT_* values for the different samples.

The fitted parameters values for the SLM samples are displayed in Figure 8, highlighting the high deviation that shows both parameters, *R_CT_* and *C_DL_*, for all samples, which can be attributed to the presence of the porosity. In the same way as the hardness tests, the samples that exhibited a higher resistance are those manufactured with intermediate energy density. The low *R_CT_* values are correlated with defects in AM specimens [30,37]. In addition, the lower values of the double-layer capacity correspond with the samples that present a higher resistance. The high *C_DL_* values for the H122 and I138 samples are attributed to defects, including non-homogenous aspects of the surface and presence of pores [44]. Since the capacity is correlated with the active surface of the sample, representations have been made versus porosity (not included). However, no clear trend has been obtained for low porosities, that is, for samples manufactured with medium energy densities. These results is in good agreement with those published by Sander et al. [23] and Revilla et al. [22], who indicated that the electrochemical behavior was not affected in samples with low porosity. The values of resistance and capacitance are consistent with previous studies, with similar values to Revilla et al., who obtained resistances between 2.1–2.9 MΩ·cm^2^ [22]. However, the impedance values are higher than those indicated by Kale et al. with resistances values of about 16–450 kΩ·cm^2^ [30] and Lodhi et al., who reported values of 600 kΩ·cm^2^ in a chlorinated medium [26].

**Figure 9:**
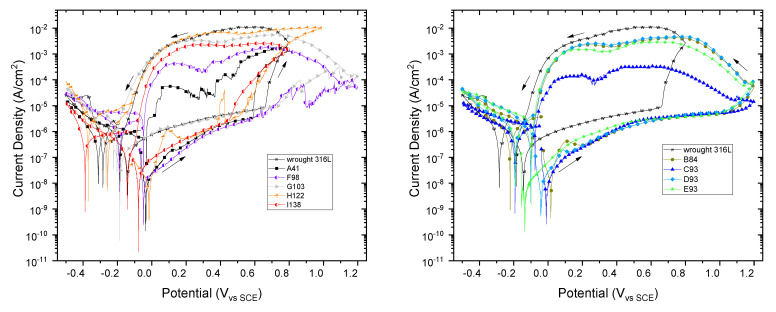
Cyclic voltammetries for SLM 316L SS specimens in 3.5 wt% NaCl. (CV of wrought 316L is included as reference).

**Figure 10:**
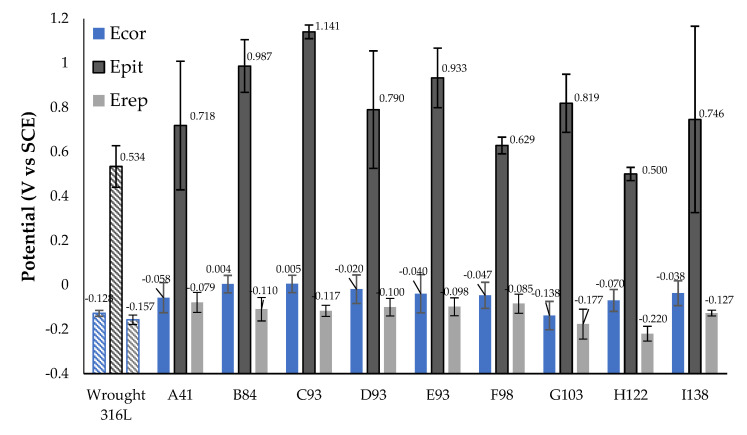
Ecorr, Epit and Erep values obtained from CVs of SLM 316L CV and wrought 316L. Error bars represent the standard deviations.

Figure 9 shows the cyclic voltammetries (CVs) for SLM 316L SS samples. The CV for wrought 316L is included as a reference. The CVs exhibit a similar morphology, highlightsing the wide range of passivity potentials and the low current densities of the SLM samples. The corrosion potential (Ecorr), pitting potential (Epit) and repassivation potential (Erep) obtained from the CVs for all samples are displayed in Figure 10. The error bar represents the standard deviation of the three replicates. As the impedance measurements, the potentials show a certain variability, which is common in additive manufacturing samples [23]. The highest potentials, Ecorr, Epit and Erep, correspond to the samples manufactured with energy densities close to 90 J/mm^3^ with Epit above 1 V vs. SCE and repassivation potentials close to −0.1 V. On the other hand, the higher passivity of wrought material could be due to a more stable native oxide film [22]. 

By analyzing these results in detail, it can be seen that the sample with the best corrosion behavior is C93, which shows an Epit of 1.15 Vvs SCE and a lower current density throughout the potentials range. This result is in good agreement with previous studies that show the best results with an Epit of about 1 V vs. SCE [21,22,25,26]. The lower variability of the Epit results obtained for the C93 sample may be related to the stability limit of chromium oxide. As indicated by Sun et al. [21] the SLM manufacturing allows the ultimate corrosion resistant potential of 316L to be reached at about 1.2 V vs. SCE, that is around the transpassive state of chromium oxide.

It should be noted that, although the C93, D93 and E93 samples are obtained with practically the same energy density, the potentiodynamic test reveals a great reduction in the current density of the C93 sample. Although there are no significant differences in hardness and porosity, the selection of the manufacturing parameters considering the electrochemical results allows a significant improvement in the corrosion resistance properties of SLM 316L SS. These results are in good agreement with Sander et al. [23] who did not find a significant relationship between porosity and Epit, Ecorr and icorr for samples with a low porosity. Following the same experimental design, the representation of the potentials against the porosity (not included) does not show any correlation for the samples manufactured with intermediate energy densities.

**Figure 11:**
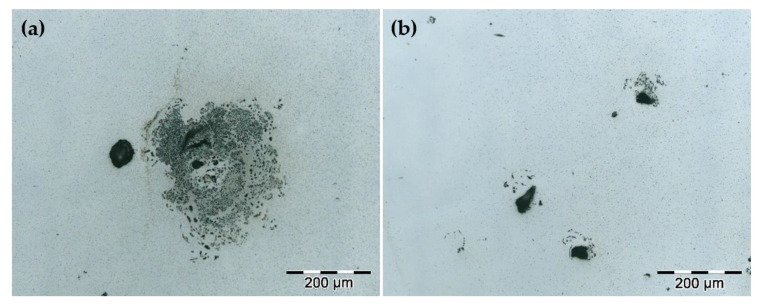
Optical micrographs of the surface of H122 after electrochemical test showing the pit morphology. (**a**) Pit with a lacy metal cover morphology; (**b**) Initial stage of the pits.

The surfaces of the corroded samples were analyzed by optical microscopy and SEM. Due to the wide sweep of the applied potentials, most of the pits were large since, after the passive layer breaks, corrosion continued in these areas, which provoked the pitting growth. Sample H122 stands out for its large number of pits compared to the other samples, which is consistent with the worst electrochemical results. Figure 11 depicts the optical micrographs corresponding to the surface of the H122 sample with a lacy metal cover morphology that is common in localized attacks on stainless steels [45]. Figure 11b shows the initial pit stage and suggests that the pit is initialized around the manufacturing defects.

**Figure 12:**
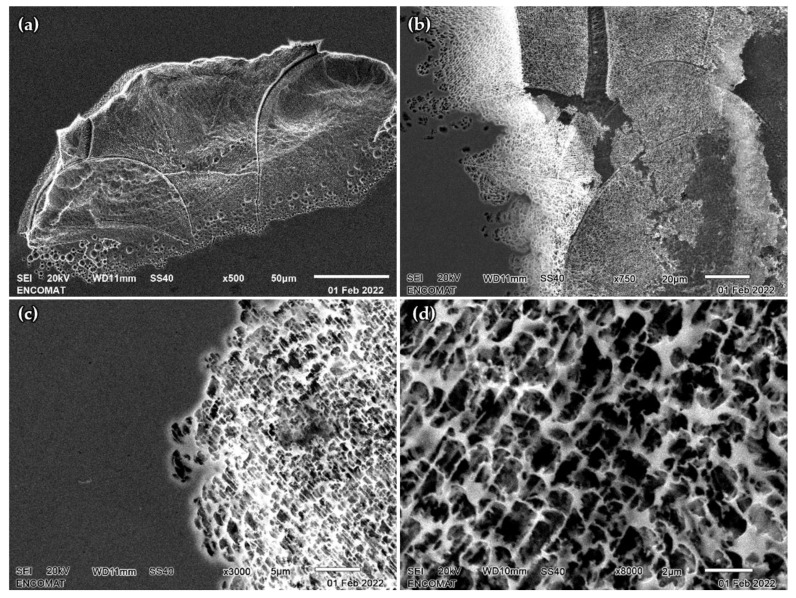
Representative SEM image micrographs of SLM 316L SS surfaces after CV. (**a**) Large pit showing preferential corrosion at the boundaries of the melt pools. (**b**–**d**) Porous structure covering the corroded area at different magnifications.

The representative SEM micrographs of surfaces of the SLM 316L SS samples after CV are displayed in Figure 12. The corroded areas reveal the presence of the melt pools, which are perfectly defined, and a preferential increase in corrosion takes place on the boundaries of the melt pools (Figure 12a). Some pits reveal the presence of a porous structure that covers the corroded area, as can be seen in Figure 12b. This structure is similar to the cellular structure described in microstructural characterization section, and the dimensions perfectly match the range of the cell size. Therefore, it is reasonable to believe that this porous structure corresponds to the boundaries of the cells that have not dissolved during CV. The corrosion process has a similar effect to an etching agent but is more selective.

**Figure 13:**
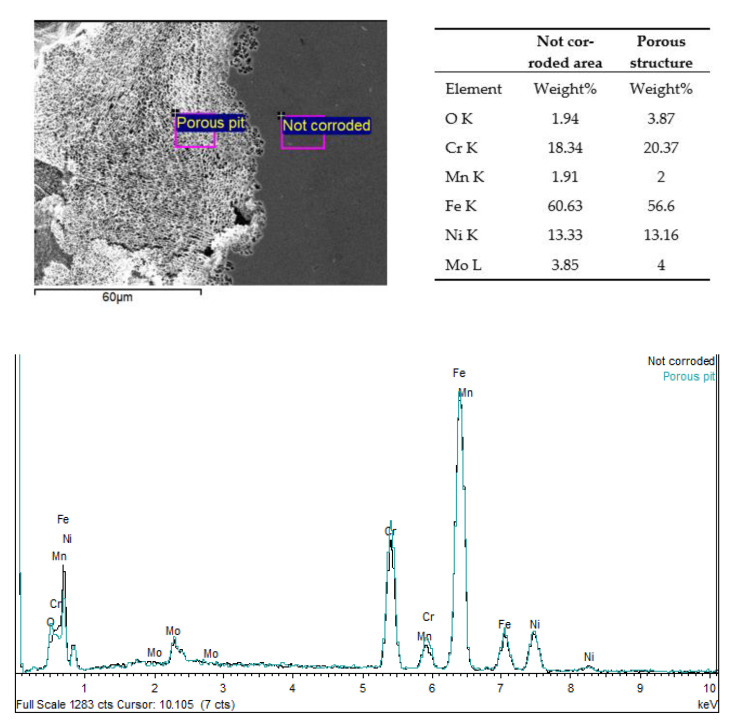
SEM image of the corroded area of C93 sample, indicating the areas where the EDS analyses were performed. Additionally, the EDS spectra of the porous pit in C93 sample are included.

To deepen the analysis of the preferential progress of corrosion, the composition of the corroded zone and the porous structure were compared. Figure 13 shows the EDS analysis of both areas that correspond to C93 sample. The differences in composition are not significant, but a higher concentration of Cr and Mo is observed in the corroded area with a porous structure. This corroborates the separation of both elements at the cell boundaries [35,46], although some references indicate that this separation is responsible for the worse electrochemical behavior of SLM samples [35]. We observed a great improvement in the selected manufacturing conditions, which reached pitting potentials close to the stability limit of the chromium oxide.

## 4. Conclusions

The effect of the laser speed (in the range 400–1200 mm/s) and hatch distance (in the 0.11–0.2 mm) on the corrosion properties of SLM 316L was optimized considering the electrochemical results of nine samples. 

The best results were obtained for an intermediate input energy density (92.5 J/mm^3^), small hatch distance (0.16 mm) and medium laser speed (625 mm/s). These manufacturing conditions produce the highest Epit and a very low current density throughout the scan potentials. 

Although the results of the microstructural and mechanical characterization allow for the selection of suitable building parameters with low porosity (<0.2%) and high hardness values, it is important to take into account the electrochemical analysis to improve the corrosion resistance.

It should be noted that, despite obtaining good electrochemical results for other samples manufactured with a very close energy density, (about 93 J/mm^3^) the small variation in the parameters maximizes the corrosion resistance.

The segregation of Cr and Mo at the cell boundaries was identified by the characteristic progress of corrosion. Despite of the presence of segregation, the electrochemical response obtained for best manufacturing conditions is much higher than that of wrought 316L.

## Figures and Tables

**Table 1:** Summary of the bibliographic results related to the corrosion behavior of 316L manufactured by SLM.

Ref.	Laser Power (*P*) (W)	Scanning Speed (*v*) (mm/s)	Hatch Spacing *(h*) (μm)	Layer Thickness (*t*) (μm)	Energy Density ^1^ (*E*) (J/mm^3^)	Corrosion Results	Grinding Level	Relative Density
[20]	175	730	120	30	66.6	SLM Epit: 740 mV vs. SCE. Passivity > wrought 316L (0.6 M NaCl)	0.04 μm colloidal silica	-
[21]	-		-	--	-	SLM Epit 700 mV > wrought 316L (0.9 wt% NaCl)	#800 grit SiC	99.4%–99.6%
[22]	180	600	20	25	600	SLM Epit 900 mV > wrought 316L (3.5 wt% NaCl)	1 μm surface finishin	-
[23]	165–285	860–1160	110	40	39.1–75.3	SLM Epit 300 mV > wrought 316L (NaCl 0.1M solution)	1 μm	-
[24]	90	1000	105	30	28.6	SLM Epit > wrought 316L (high variability) (NaCl 0.1 M solution)	-	-
[25]	180	600	36	25	333	SLM Epit = 1.4 V vs. Ag/AgCl (3 M KCl) >> wrought 316L (3.5 wt% NaCl)		-
[26]	200	-	100	30	-	SLM Epit = 920 mV vs. SCE >> wrought 316L (0.9 M NaCl)	1200 grit SiC	-
[27]	150	800–1400	25	25	171–300	SLM Epit 300 mV > wrought 316L (3.5 wt% NaCl)	2000 grit SiC	-
[28]	285	960	110	40	67	SLM Epit 100 mV > wrought 316L (3.5 wt% NaCl)	-	-
[29]	200	800	-	120		SLM Epit 200 mV > wrought 316L (simulated body fluid	2000 grit SiC	-
[30]	200	800	100	30	83.3	SLM ≈ Epit vs. wrought 316L (3 wt% NaCl solution)	2400 grit SiC	99.58%
[31]	150–200	300–600	10–50	50	100–1333	SLM Epit: 560 mV vs. Ag/AgCl, ≈ wrought 316L Ringer’s solution pH 6.9 ± 0.2.	0.04 µm Colloidal silica	98%
[32]	200	-	100	30	-	SLM Epit < wrought 316L pH (1, 2 and 3)	1 μm diamond suspension	-
[33]	150	125–200	90	50	167–267	SLM Epit < wrought 316L (0.9 wt% NaCl)	1200 grit SiC	98.3%
[34]	195	1083	25	25	288	SLM < wrought 316L 0.5 M H_2_SO_4_, 50 ppm Cl^−^ and 2 ppm F^−^	2000 grit SiC	-
[35]	200	590	50	50	135.6	SLM 316L: low passivity and high anodic current (0.1 M HCl)	0.25-µm diamond suspension	-

^1^ Calculated taking into account Equation (1) (E=P/(v·h·t).

**Table 2:** Chemical composition (wt%) of the EOS 316L stainless steel powder.

Cr	Ni	Mo	C	Cu	Mn	N	P	S	Si	Fe
17–19	13–15	2.25–3	0.03	0.5	2	0.1	0.025	0.01	0.75	Balance

**Table 3:** The SLM parameters used for manufacturing the different 316L SS specimens. Laser power was kept fixed at 370 W and the layer thickness was 0.04 mm.

Sample	A41	B84	C93	D93	E93	F98	G103	H122	I138
Laser speed (mm/s)	1200	550	625	500	555	496.74	450	400	608
Hatch distance (mm)	0.19	0.2	0.16	0.2	0.18	0.19	0.2	0.19	0.11
E (J/mm^3^)	40.57	84.09	92.5	92.5	92.59	98.01	102.78	121.71	138.31

**Table 4:** The porosities of SLM 316L samples determined using optical microscopy.

**Sample**	**A41**	**B84**	**C93**	**D93**	**E93**	**F98**	**G103**	**H122**	**I138**
Porosity (%)	0.46	0.28	0.16	0.17	0.22	0.26	0.53	0.54	0.65
